# Five Dinuclear Lanthanide Complexes Based on 2,4-dimethylbenzoic Acid and 5,5′-dimethy-2,2′-bipyridine: Crystal Structures, Thermal Behaviour and Luminescent Property

**DOI:** 10.3389/fchem.2021.726813

**Published:** 2021-10-18

**Authors:** Jia-Yuan Zhao, Ning Ren, Ying-Ying Zhang, Kun Tang, Jian-Jun Zhang

**Affiliations:** ^1^ College of Chemistry and Materials Science, Testing and Analysis Center, Hebei Normal University, Shijiazhuang, China; ^2^ Hebei Key Laboratory of Heterocyclic Compounds, College of Chemical Engineering and Material, Handan University, Handan, China; ^3^ Hengshui No.1 High School, Hengshui, China; ^4^ Huaxin College of Hebei Geo University, Shijiazhuang, China

**Keywords:** lanthanide complexes, aromatic carboxylic acids, TG/DSC-FTIR, crystal structure, luminescence

## Abstract

A series of new complexes, [Ln (2,4-DMBA)_3_(5,5′-DM-2,2′-bipy)]_2_ (Ln = Sm(1), Eu (2)), [Pr (2,4-DMBA)_3_ (5,5′-DM-2,2′-bipy)]_2_·0.5(C_2_H_5_OH) (3), [Ln (2,4-DMBA)_3_ (5,5′-DM-2,2′-bipy)]_2_·0.5(2,4-DMBAH)·0.25(5,5′-DM-2,2′-bipy) (Ln = Tb (4), Dy (5)) (2,4-DMBA = 2,4-dimethylbenzoate, 5,5′-DM-2,2′-bipy = 5,5′-dimethy-2,2′-bipyridine) were synthesized via hydrothermal reaction conditions. The complexes were characterized through elemental analysis, Infrared spectra (IR), Raman (R) spectra, UV-Vis spectra, single X-ray diffraction. Single crystal data show that complexes **1**–**5** are binuclear complexes, but they can be divided into three different crystal structures. The thermal decomposition mechanism of complexes **1**–**5** were investigated by the technology of simultaneous TG/DSC-FTIR. What’s more, the luminescent properties of complexes **1**–**2** and **4** were discussed, and the luminescence lifetime (τ) of complexes **2** and **4** were calculated.

## Introduction

In recent years, the synthesis and construction of metal complexes and supramolecular complexes have attracted extensive attention (Feng et al., 2010). Metal complexes have potential applications in catalysis (Mikami et al., 2002), gas adsorption (Wang et al., 2010), magnetism (Duan et al., 2020; Xi et al., 2020), fluorescence (Kuzmina et al., 2018) and so on. Lanthanide ions have unique 4f electronic structure, which leads to long luminescence lifetimes, narrow luminescence emission peaks and large Stokes shifts (Zheng et al., 2019). Therefore, lanthanide ions with special electronic structure are preferred in the selection of metal ions, which makes the complexes have excellent optical properties and can be used in the fields of luminescent materials, biological imaging, biological response probes and sensors (Liu et al., 2014a; Abbas et al., 2017; Gu et al., 2018). Moreover, lanthanide ions as metal center ions have the advantages of large radius, high coordination number and diverse coordination environment (Soek et al., 2019). In addition to the selection of lanthanide ions, the suitable ligands are also very critical. Among many organic ligands, aromatic carboxylic acid ligands are generally considered to be excellent organic ligands due to their ability to provide oxygen atoms and affinity for lanthanide cations (Su et al., 2012). In addition, aromatic carboxylic acid ligands have multiple coordination sites, and oxygen atoms of carboxylic acid ligands can coordinate with lanthanide ions in various coordination modes, so lanthanide organic coordination complexes with various structures can be constructed (Li et al., 2019). Most importantly, the introduction of aromatic carboxylic acid ligands can sensitize the fluorescence of lanthanide ions. Because the 4f-4f transition is forbidden by spin and parity, the luminescence intensity of lanthanide ions is relatively weak. It is well known that the antenna effect is a popular method to improve the luminescent properties of lanthanide elements. It includes the effective absorption of incident light through organic chromophores and the corresponding sensitization of lanthanide metal ions through ligand to metal energy transfer (Ahmed and Iftikhar, 2010; Fomina et al., 2012; Carter et al., 2016). On the other hand, bipyridyl ligands also become one of the attractive ligands because of their strong coordination ability and conjugated large π system. The introduction of nitrogen-containing ligands can also improve the chemical and thermal stability of the complexes (Cai et al., 2019). The study of thermal stability, decomposition mechanism and kinetics of the complexes can provide important reference for the synthesis of functional materials with certain thermal stability (Zong et al., 2015; El-Enein et al., 2019; Ren et al., 2020; Wang and Zhang, 2020; Zhou et al., 2021). Therefore, we choose 2,4-dimethylbenzoic acid as acid ligand and 5,5′-dimethy-2,2′-bipyridine as nitrogen-containing neutral ligand to synthesize lanthanide complexes.

In this paper, five lanthanide complexes have been successfully prepared. We report the synthesis, crystal structure, thermal behaviour and luminescence property of complexes **1**–**5**. Single crystal data show that complexes **1**-**5** are binuclear complexes, but they can be divided into three different crystal structures. Complexes **1**-**3** form 1D chain structure, while complexes **4**-**5** form 2D sheet structure. The thermal behaviour of complexes **1**-**5** were studied by TG-DSC/FTIR. At the same time, we also discussed the luminescence behavior of complexes **1**-**2** and **4**, and the luminescence lifetime (τ) of complexes **2** and **4** were calculated.

## Experimental

### Materials and Reagents

Ln (NO_3_)_3_·6H_2_O, 2,4-dimethylbenzoic acid and 5,5′-dimethyl-2,2′-bipyridine were are commercially available and can be used as supplied without processing.

Elemental analysis (C, H, N) were performed with a Vario-EL II elemental analyzer. The IR spectra were measured by a Bruker TENSOR 27 spectrometer using KBr pellets in the range of 4,000 cm^−1^ to 400 cm^−1^. The ultraviolet spectra measurements were carried out on a SHIMADZU 2501 spectrometer. The Raman spectra were recorded by a Bruker VERTEX-70 FTIR-RAMANII instrument over the range of 50–4,000 cm^−1^, and scanned 64 times. The TG/DSC-FTIR tests were run on a NETZSCH STA 449F3 instrument under a dynamic atmosphere of air with a heating rate of 10 K min^−1^. The photoluminescence spectra and lifetime were measured on FS5 spectrofluorophotometer.

### Synthesis

The weighed 2,4-dimethylbenzoic acid (0.6 mmol) and 5,5′-dimethy-2,2′-bipyridine (0.2 mmol) were mixed and dissolved in 7 ml 95% ethanol solution. After the solution was completely dissolved, the pH of the solution was adjusted to 5-7 through adding NaOH (1 mol/L) solution. Then the mixed ligands solution was dripped into 3 ml aqueous solution of Ln (NO_3_)_3_·6H_2_O (0.2 mmol) under stirring. After stirring for 40 min, the mixture was placed into a 25 ml Teflon-lined autoclave and heated at 393.15 K for 7 d. Element analysis for C_78_H_86_N_4_O_16_Sm_2_ (%),Calcd: C, 57.26; H, 5.30; N, 3.42; Found: C, 57.14; H, 5.14; N, 3.51; C_78_H_82_N_4_O_14_Eu_2_ (%),Calcd: C,58.43; H, 5.16; N,3.49; Found: C, 58.62; H, 5.16; N, 3.57; C_79_H_81_N_4_O_12.5_Pr_2_ (%),Calcd: C,60.50; H, 5.21; N,3.57; Found: C, 60.65; H, 5.26; N, 3.69; C_85.5_H_86_N_4.50_O_13_Tb_2_ (%),Calcd: C,60.32; H, 5.09; N,3.70; Found: C, 60.12; H, 5.14; N, 3.63; C_85.5_H_86_N_4.50_O_13_Dy_2_ (%),Calcd: C,60.07; H, 5.07; N,3.69; Found: C, 60.10; H, 5.13; N, 3.54.

### X-Ray Structure Solution and Refinement

All the crystallographic data for complexes **1-5** were collected on a Smart-1000 diffractometer with graphite-monochromated Mo-Kα radiation (*λ* = 0.71073 Å) at room temperature. The structures of all complexes were solved using direct methods and refined by full-matrix least squares on F^2^ of SHELXS-97 program. Additionally, the solvate molecules in complexes **1** and **2** are severely disordered. Attempts to model the disorder were not very successful but were replaceed by SQUEEZE (Spek, 2009; Kose et al., 2019). Squeeze located one void containing 40 electrons in the unit cell for complex **1**. Combined with element analysis and thermogravimetric analysis, this was interpreted as four water molecules (40 electrons) per cell. Similarly, the disorder solvent molecule for complex **2** is interpreted as two water molecules.

## Results and Discussion

### FT-IR Spectra

**FIGURE 1:**
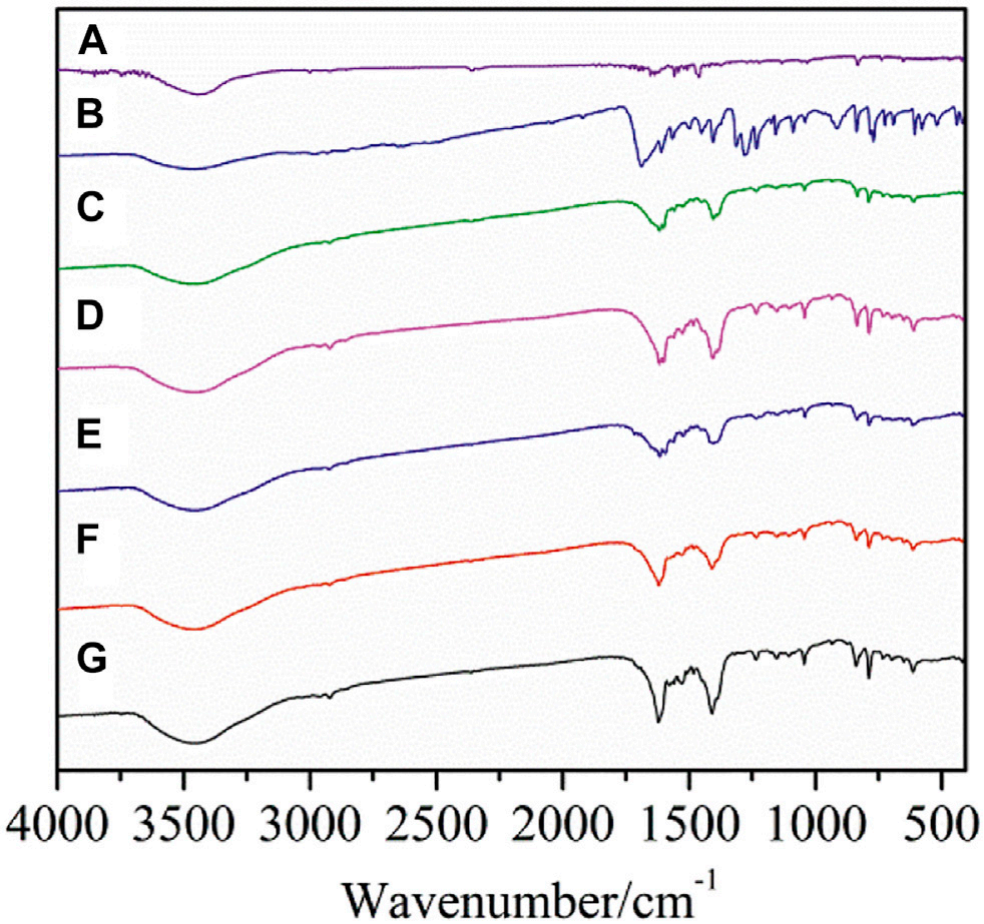
FT-IR spectra of the complexes and ligands (A: 5,5′-DM-2,2′-bipy, B: 2,4-DMBA, C–G: complexes **1**–**5**).

The data of the characteristic absorption bands in the IR spectra of the two crystal complexes and the ligands were shown in Supplementary Table S1. As shown in Figure 1, the IR spectra of complexes **1**-**5** are different from those of the two ligands, illustrating that new complexes have been formed. The characteristic absorption peak of carboxylic acid group ν_C=O_ at wave number 1693 cm^−1^ of the 2,4-dimethylbenzoic acid ligand disappeared in the formation of the complexes. At the same time, antisymmetric ν_as(COO_^−^_)_ and symmetric ν_s(COO_^−^_)_ stretching vibration absorption peaks are observed at 1,605–1,615 cm^−1^ and 1,411–1,422 cm^−1^. In addition, Ln-O vibration absorption peak appears at 418–419 cm^−1^. These changes indicate that carboxylic acid ligands are coordinated with Ln^3+^ ions. The ν_C=N_ stretching vibration absorption peak of neutral ligand 5,5′-DM-2,2′-bipy at 1,588 cm^−1^ shows a obvious shift after the formation of the complex, indicating that the 5,5′-DM-2,2′-bipy ligands are coordinated with Ln^3+^ ions (Li et al., 2017).

### UV-Vis Spectra

The UV-Vis spectra of the complexes **1**–**5**, 2,4-dimethylbenzoic acid and 5,5′-dimethy-2,2′-bipyridine are measured in the concentration of 1 × 10^–5^ mol/L using DMSO as solvent and the data are listed in Table 1.

**TABLE 1:** UV-Vis spectra data of ligands and complexes.

Ligands/complexes	λ_max_/nm	A_max_
2,4-dimethylbenzoic acid	274	0.3238
5,5′-dimethyl-2,2′-bipyridine	291	0.3019
1	290	0.9741
2	290	0.6363
3	290	1.0557
4	288	0.8356
5	288	1.2334

It can be seen from the table that the complexes and ligands have different degrees of absorption in the ultraviolet region, and the positions and intensities of the absorption peaks are different. The absorption bands of the ligands 2,4-dimethylbenzoic acid and 5,5′-dimethy-2,2′-bipyridine are found at 274 and 291 nm, respectively, which are assigned to the π→π* transition of benzene skeleton. After formation of the complexes, the absorption bands shift to 288–290 nm, which indicates that 2,4-dimethylbenzoic acid and 5,5′-dimethy-2,2′-bipyridine ligands coordinate to the Ln^3+^ ions in the complexes. At the same time, the maximum absorbances (Amax) of the complexes are obviously higher than that of the ligands, indicating that the complexes have a larger conjugation system (Taha et al., 2017).

### Raman Spectra

**FIGURE 2:**
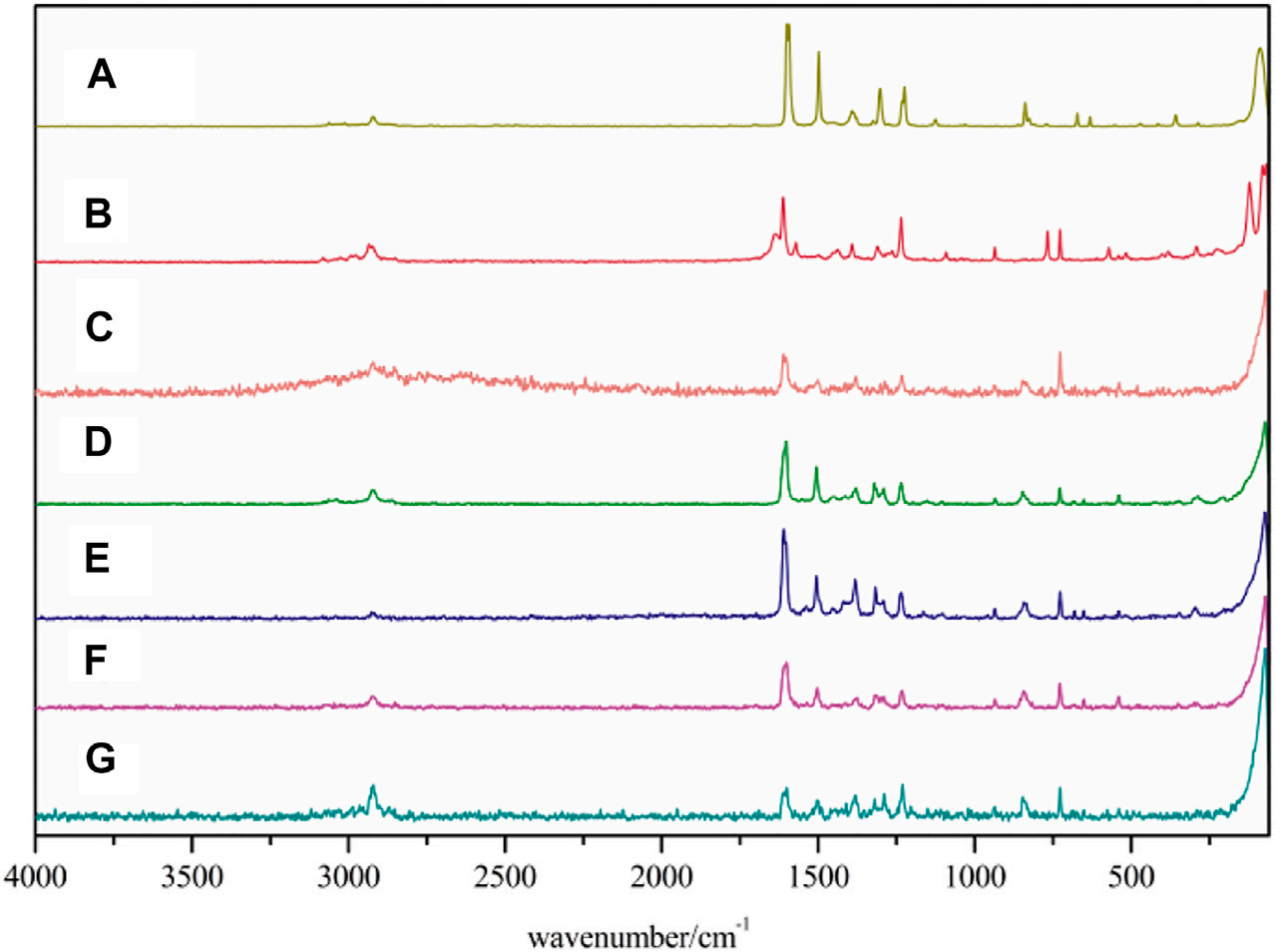
Raman spectra of the complexes and two ligands (A: 5,5′-DM-2,2′-bipy, B:2,4-DMBA, C–G: complexes **1**–**5**).

The Raman spectra are also used to characterize lanthanide complexes, and also provide information about the molecular structure. The Raman spectra of both the complexes and ligands are shown in Figure 2. The Raman spectra data of ligands and complexes are listed in Supplementary Table S2. The stretching vibrations of 2,4-dimethylbenzoic acid is corresponded to the strong Raman bands at 1,612 cm^−1^. When the complexes are formed, the bands disappear while the asymmetric and symmetric stretching vibrations of COO^−^ appear at 1,601–1,611 cm^−1^ and 1,501–1,506 cm^−1^, respectively. For 5,5′-dimethy-2,2′-bipyridine ligand, the band at 1,497 cm^−1^ is assigned to the stretching vibration peaks of C=N. The band shifted to 1,375–1,382 cm^−1^ in the complexes **1**-**5**, indicating that nitrogen atoms are coordinated with the metal ions. In the complexes, the bands at 325–351 cm^−1^ and 257–291 cm^−1^ can be attributed to the Ln-O and Ln-N stretching vibration, respectively. These results indicate that the two ligands are coordinated with Ln^3+^ ions (Huang et al., 2012; He et al., 2014).

### Crystal Structure

The crystal structure of complexes **1**-**5** was determined by single crystal X-ray diffraction. The results show that the series of complexes have three different structural types. Complexes **1**–**2** (structure type Ⅰ) are isomorphic and crystallized in the triclinic space group Pī. Complex **3** (structure type Ⅱ) crystallized in the monoclinic space group C2/c. Complexes **4**–**5** (structure type Ⅲ) crystallized in the triclinic space group Pī. Complexes **1**-**2** and **4**-**5** have the same crystal system and space group, but they have different coordination number and coordination environment. The crystallographic data of complexes **1**-**5** are listed in Table 2 and the corresponding bond lengths are listed in Supplementary Tables S3, S4.

**TABLE 2:** The crystal data and structure refinement for the complexes.

Complexes	1	2	3	4	5
Empirical formula	C_78_H_78_N_4_O_12_Sm_2_	C_78_H_78_N_4_O_12_Eu_2_	C_158_H_162_N_8_O_25_Pr_4_	C_85.5_H_86_N_4.50_O_13_Tb_2_	C_85.5_H_86_N_4.50_O_13_Dy_2_
Formula weight	1,564.14	1,567.36	3,136.60	1702.43	1709.59
Temperature/K	298 (2)	298 (2)	298 (2)	298 (2)	298 (2)
Wavelength/Å	0.71073	0.71073	0.71073	0.71073	0.71073
Crystal system	Triclinic	Triclinic	Monoclinic	Triclinic	Triclinic
Space group	P ī	P ī	C2/c	P ī	P ī
*a*/Å	12.8743 (11)	12.6627 (12)	27.569 (3)	11.7300 (11)	11.8155 (11)
*b*/Å	15.7404 (12)	15.4402 (14)	12.4586 (11)	12.8949 (12)	13.0099 (12)
*c*/Å	22.5043 (19)	22.0986 (19)	24.245 (2)	16.1841 (15)	16.2994 (14)
*α/*°	94.957 (2)	95.1490	90	108.142 (3)	108.189 (3)
*β/*°	104.740 (3)	104.757 (2)	117.479 (3)	109.849 (3)	109.813 (3)
*γ/*°	111.108 (4)	111.217 (3)	90	95.145 (2)	95.235 (2)
Volume/Å^3^	4,033.5 (6)	3,814.7 (6)	7388.0 (11)	2135.1 (3)	2184.3 (3)
*Z,* Calculated density/mg m^−3^	2, 1.288	2, 1.365	2, 1.410	1, 1.324	1, 1.300
Absorption coefficient/mm^−1^	1.498	1.689	1.367	1.702	1.756
*F* (000)	1,588	1,592	3,204	865	867
Crystal size/mm	0.15 × 0.05 × 0.04	0.20 × 0.08 × 0.04	0.40 × 0.12 × 0.04	0.22 × 0.10 × 0.04	0.20 × 0.14 × 0.09
Theta range for data collection/deg	2.14 to 25.02	2.18 to 25.02	2.34 to 25.02	2.46 to 25.02	2.44 to 25.02
Limiting indices	−13 ≤ *h* ≤ 15	−15 ≤ *h* ≤ 10	−30 ≤ *h* ≤ 32	−13 ≤ *h* ≤ 13	−12 ≤ *h* ≤ 14. −15 ≤ *k* ≤ 15. −19 ≤ *l* ≤ 16
	−16 ≤ *k* ≤ 18	−16 ≤ *k* ≤ 18	−14 ≤ *k* ≤ 13	−14 ≤ *k* ≤ 15	
	−26 ≤ *l* ≤ 24	−26 ≤ *l* ≤ 26	−28 ≤ *l* ≤ 26	−16 ≤ *l* ≤ 19	
Reflections collected/unique	9130/9130 [*R* (int) = 0.1270]	19,697/13,286 [*R* (int) = 0.1426]	17,488/6488 [*R* (int) = 0.0791]	10,958/7420 [*R* (int) = 0.0527]	11,153/7591 [*R* (int) = 0.0652]
Completeness to theta = 25.02	98.5%	98.6%	99.6%	98.6%	98.5%
Max. and min. transmission	0.9425 and 0.8065	0.9355 and 0.7288	0.9474 and 0.6109	0.9350 and 0.7058	0.8580 and 0.7203
Data/restraints/parameters	14,032/0/881	13,286/2892/865	6488/0/533	7420/821/596	7591/714/596
Goodness-of-fit on *F* ^*2*^	1.089	1.042	1.100	1.075	1.188
Final *R* indices [*I > 2σ(I)*]	*R*_1_ = 0.1380 w*R*_2_ = 0.3480	*R*_1_ = 0.1219 w*R*_2_ = 0.2364	*R*_1_ = 0.0584 w*R*_2_ = 0.1106	*R*_1_ = 0.0614 w*R*_2_ = 0.1367	*R*_1_ = 0.0731 w*R*_2_ = 0.1549
*R* indices (all data)	*R*_1_ = 0.1890 w*R*_2_ = 0.3744	*R*_1_ = 0.2214 w*R*_2_ = 0.2603	*R*_1_ = 0.0989 w*R*_2_ = 0.1241	*R*_1_ = 0.0936 w*R*_2_ = 0.1478	*R*_1_ = 0.1096 w*R*_2_ = 0.1660
Largest diff. peak and hole (e Å^−3^)	2.273 and −1.377	1.978 and −1.254	1.077 and −0.864	1.817 and −0.589	2.409 and −0.986

### [Eu(2,4-DMBA)_3_(5,5′-DM-2,2′-bipy)]_2_–Structure Type Ⅰ

**FIGURE 3:**
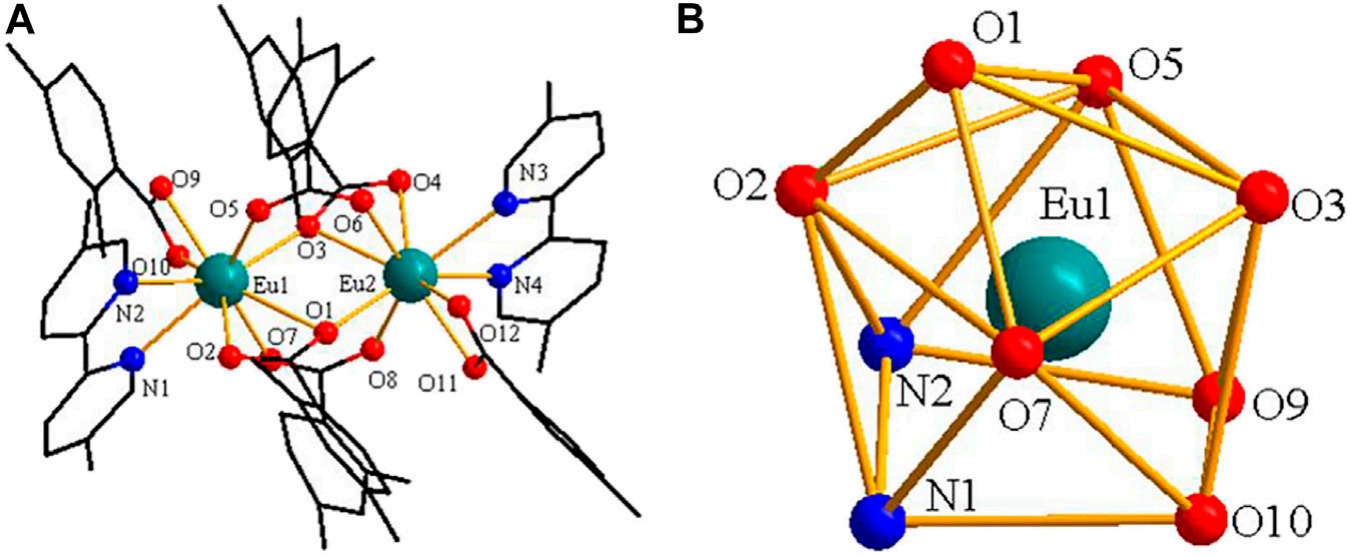
**(A)** The crystal structure of complex **2**. **(B)** The coordination polyhedron of Eu1^3+^ ion.

**FIGURE 4:**
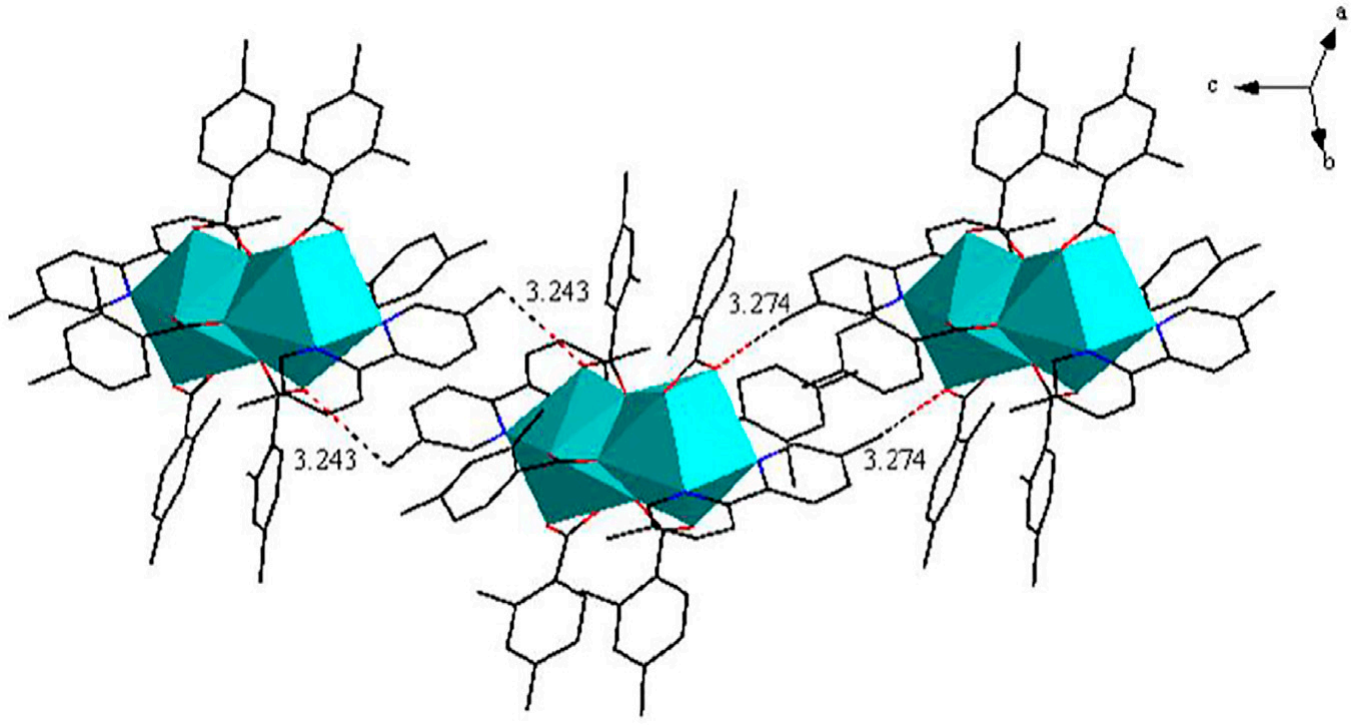
The 1D chain structure along approximately c-axis.

As shown in Figure 3A, complex **2** is a dinuclear molecule and each asymmetric unit consists of two crystallographically independent central ions (Eu1 and Eu2), six 2,4-DMBA ligands, two 5,5′-DM-2,2′-bipy ligands. The coordination number of two central Eu^3+^ ions is 9. Each center Eu^3+^ ion forms a distorted monocapped square anti-prismatic geometry environment with seven O atoms and two N atoms (Figure 3B) (Liu et al., 2013; Liu et al., 2014b). Two O atoms (O5, O7) come from two bridging bidentate 2,4-DMBA ligands; three O atoms (O1, O2, O3) come from two bridging tridentate 2,4-DMBA ligands; O9 and O10 atoms come from a chelating bidentate 2,4-DMBA ligand; and two N atoms (N1, N2) come from a 5,5′-DM-2,2′-bipy ligand. The coordination number and coordination environment of the two Eu^3+^ ions are the same, but they have different bond length and bond angle. The bond length of Eu-O around Eu1^3+^ ion is 2.334 (9)-2.688 (11) Å, and the average bond length is 2.423 Å; the average bond length of Eu-N is 2.538 Å. The bond length of Eu-O around Eu2^3+^ ion is 2.333 (10)–2.638 (10) Å, and the average bond length is 2.423 Å; the average bond length of Eu-N bond is 2.542 Å. It can be found that the average bond length between Eu-O atoms is shorter than that between Eu-N atoms. This may be because the coordination ability of O atom is stronger than that of N atom, so when the complex undergoes thermally decomposed, the neutral ligand 5,5′-DM-2,2′-bipy may always decomposes preferentially over the acid ligand 2,4-DMBA. As shown in Figure 4, a 1D chain along the crystallographic b axis is formed by the C-H···O hydrogen bonding interaction. The distance of hydrogen bonding interactions are 3.243 (Å) and 3.274 (Å).

### [Pr(2,4-DMBA)_3_(5,5′-DM-2,2′-bipy)]_2_·0.5(C_2_H_5_OH)–Structure Type Ⅱ

**FIGURE 5:**
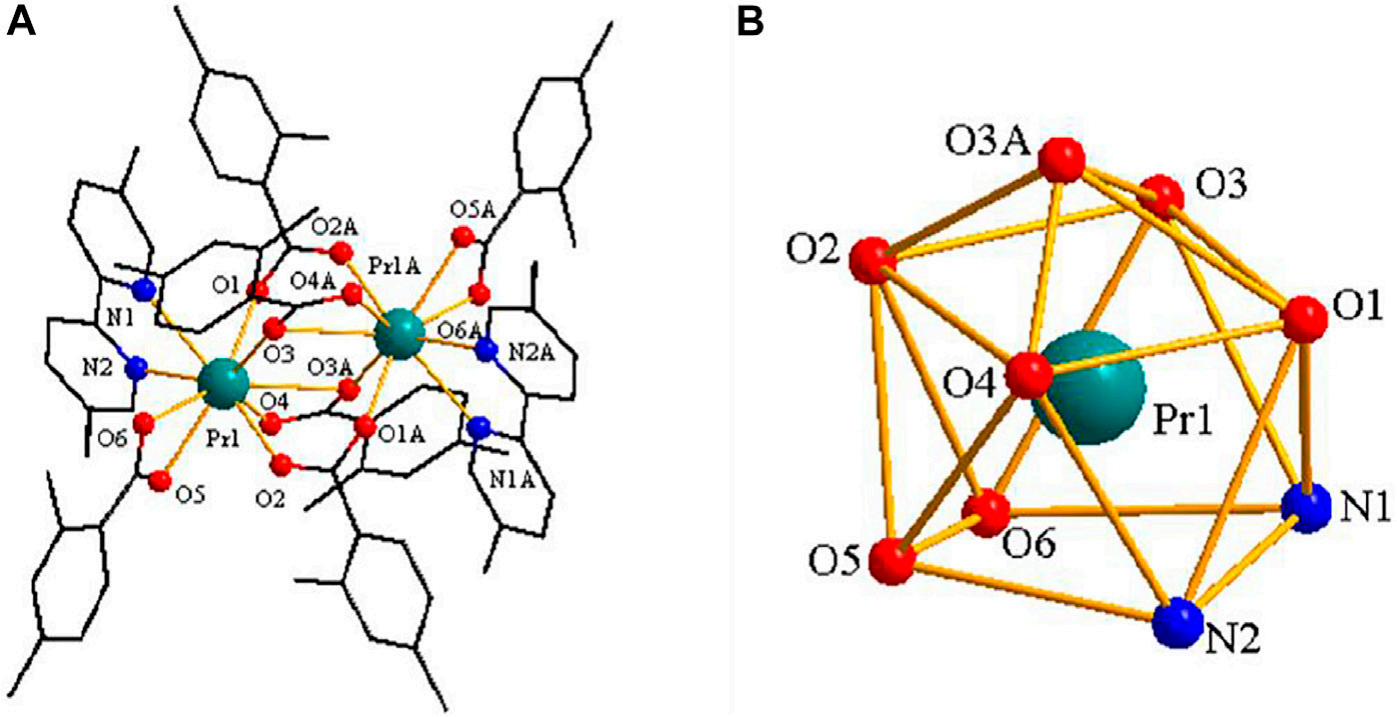
**(A)** The crystal structure of complex **3**. (the free half of methanol molecule are omitted for clarity) **(B)** The coordination polyhedron of Pr^3+^ ion.

**FIGURE 6:**
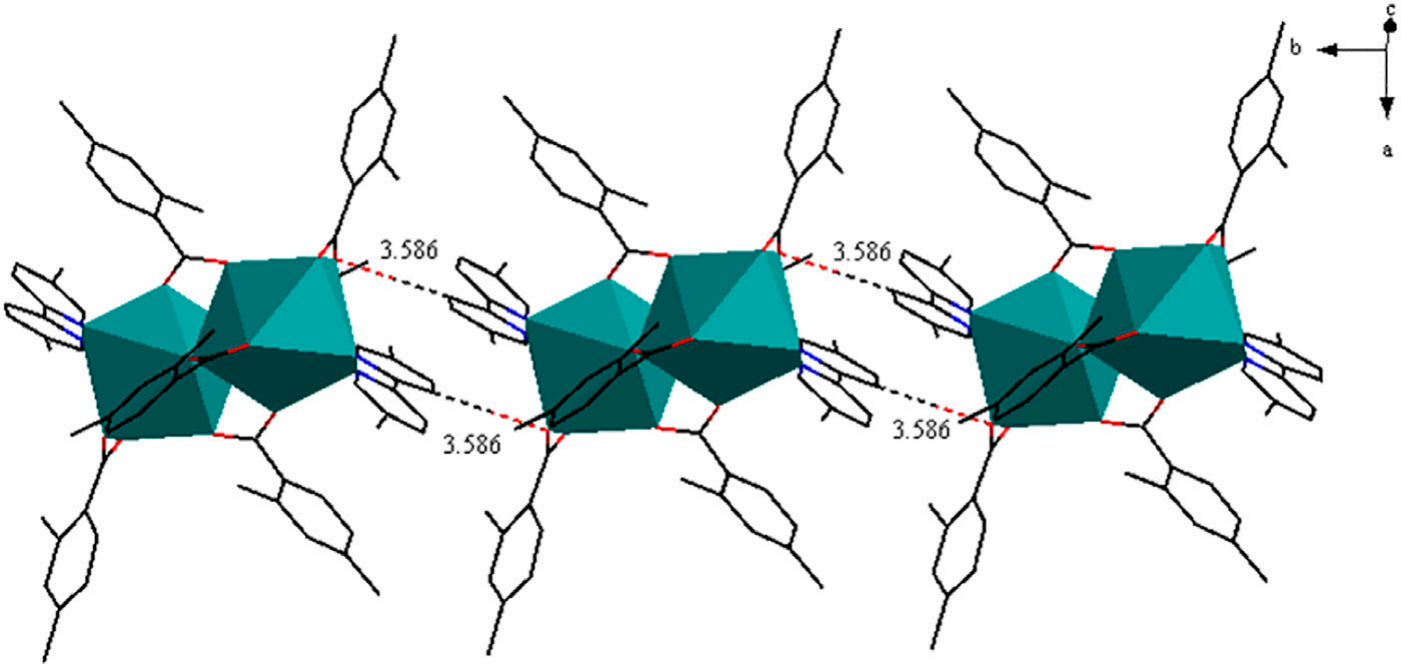
The 1D chain structure along approximately b-axis.

As shown in Figure 5A, complex **3** is composed of two Pr^3+^ ions, six 2,4-DMBA ligands, two 5,5′-DM-2,2′-bipy ligands and free half of C_2_H_5_OH molecule. Here, the coordination environment of Pr^3+^ centre is a distorted monocapped square anti-prismatic geometry (Figure 5B). The 2,4-DMBA ligands mainly adopt chelating bidentate, bridging bidentate and bridging tridentate coordination modes. Among them, O1 and O2 atoms come from two bridging bidentate 2,4-DMBA ligands; O3, O3A and O4 atoms come from two bridging tridentate 2,4-DMBA ligands; O5 and O6 atoms come from a chelating bidentate 2,4-DMBA ligand. The Pr-O average bond length is 2.527 Å whereas the average bond length of Pr-N is 2.664 Å. We can find that the average bond length of Pr-O of the complex is shorter than that of Pr-N. This may be because the coordination ability of O atom is stronger than that of N atom, so the decomposition of 5,5′-DM-2,2′-bipy is prior to that of 2,4-DMBA ligand. As shown in Figure 6, a 1D chain along the crystallographic b axis is formed by the C-H···O hydrogen bonding interaction. The distance of hydrogen bonding interactions is 3.586 (Å).

### [Ln(2,4-DMBA)_3_(5,5′-DM-2,2′-bipy)]_2_·0.5(2,4-DMBAH)·0.25(5,5′-DM-2,2′- Bipy)–Structure Type Ⅲ

**FIGURE 7:**
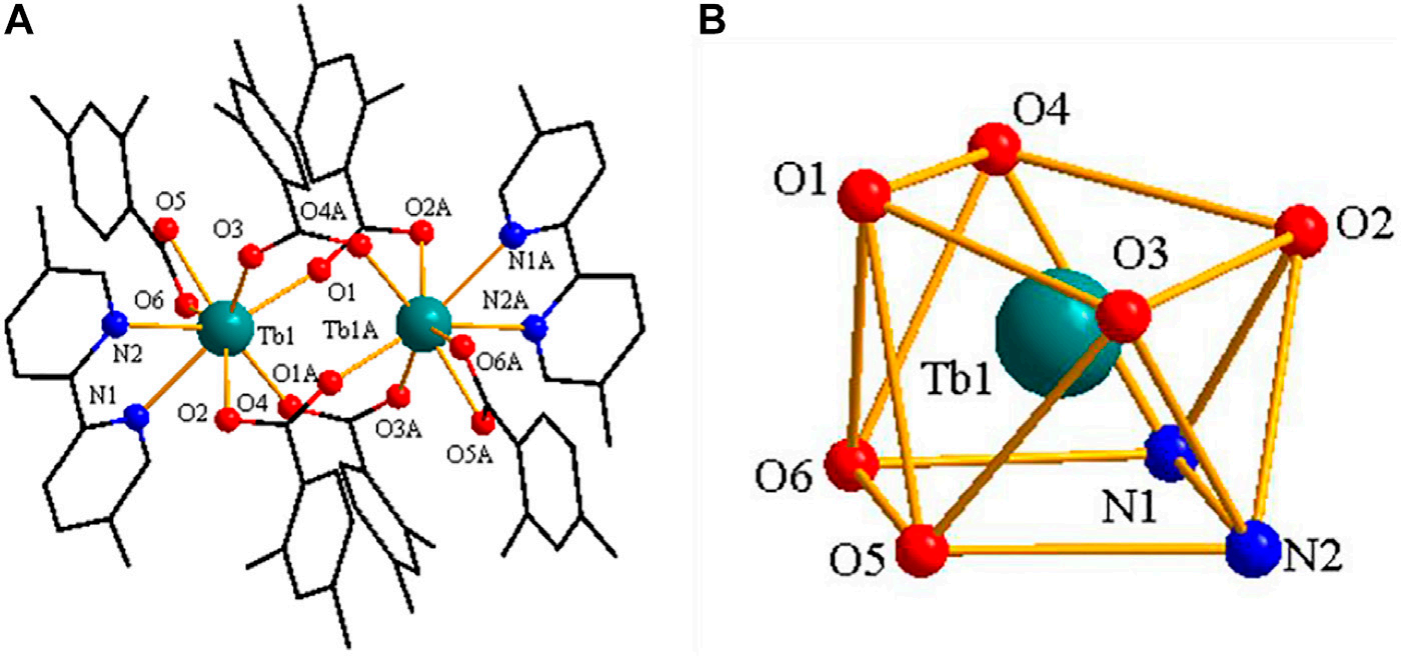
**(A)** The crystal structure of complex **3**. (the free ligand molecules are omitted for clarity) **(B)** The coordination polyhedron of Pr^3+^ ion center.

As shown in Figure 7A, the structure of complex **3** is built from 2 Tb^3+^ ions, six 2,4-DMBA ligands, two 5,5′-DM-2,2′-bipy ligands, free half 2,4-DMBAH molecule and free quarter 5,5′-DM-2,2′-bipy molecule. The coordination number of the 2 Tb^3+^ ions is 8. Each center Tb^3+^ ion has six O atoms and two N atoms in a distorted tetragonal antiprism geometry environment (Figure 7B). Four O atoms (O1A, O2, O3, O4) come from four bridging bidentate 2,4-DMBA ligands; two O atoms (O5, O6) come from a chelating bidentate 2,4-DMBA ligand; and two N atoms (N1, N2) come from a 5,5′-DM-2,2′-bipy ligand. The average bond lengths of Tb-O and Tb-N are 2.365 Å and 2.566 Å, respectively. We can find that the average bond length of Tb-O is less than that of Tb-N. Therefore, we speculate that the neutral ligand 5,5′-DM-2,2′-bipy will decompose prior to the acidic ligand 2,4-DMBA during thermal decomposition, which is consistent with the results of thermogravimetry.

**FIGURE 8:**
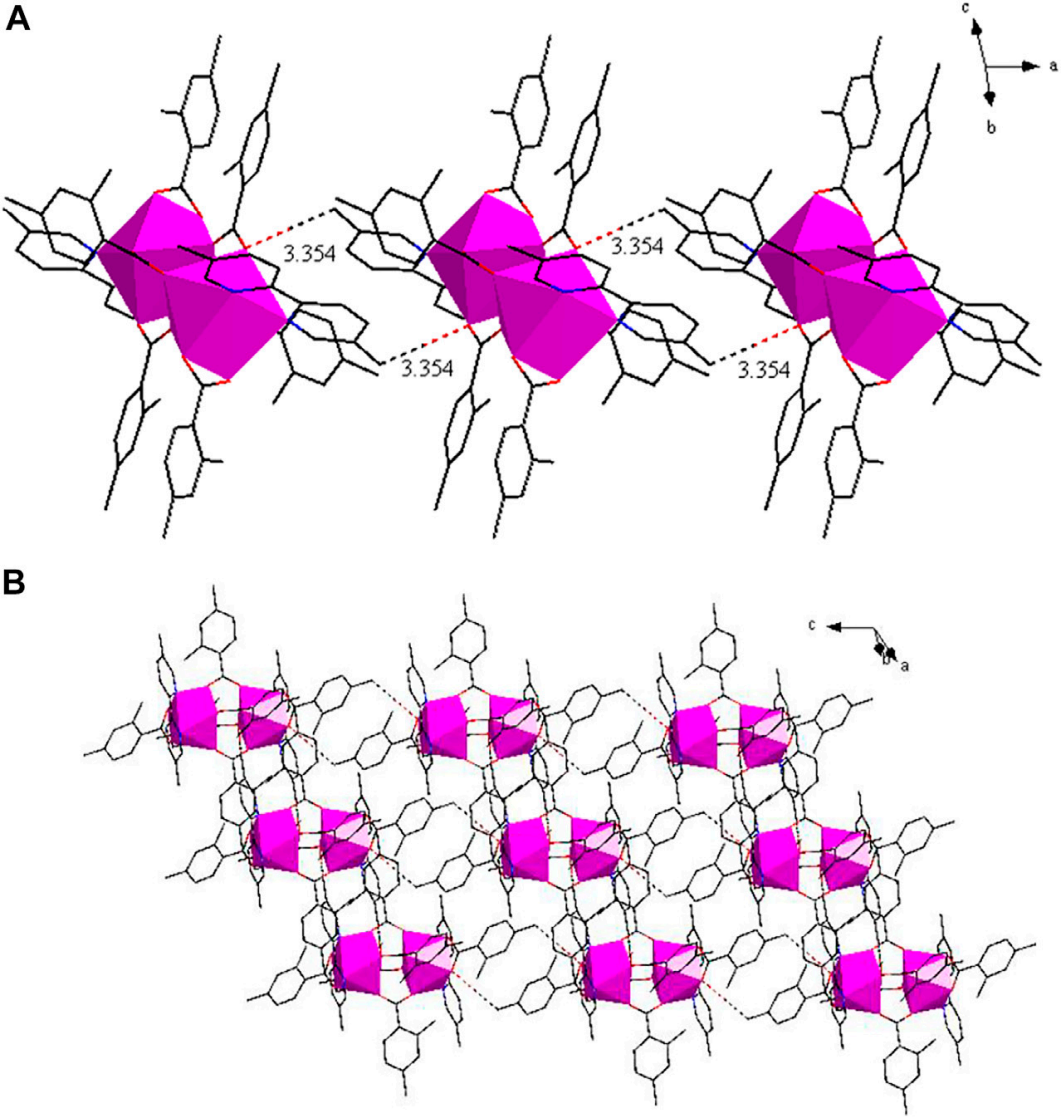
**(A)** The 1D chain structure along a-axis. **(B)** The 2D sheet in about the ac plane.

As shown in Figure 8A, two adjacent structural units through C-H···O hydrogen bonding form a 1D chain structure along along the crystallographic a axis. The adjacent infinite 1D chains through C-H···O hydrogen bonding, further assembled into a 2D sheet structure (Figure 8B).

### Thermal Decomposition Processes

**FIGURE 9:**
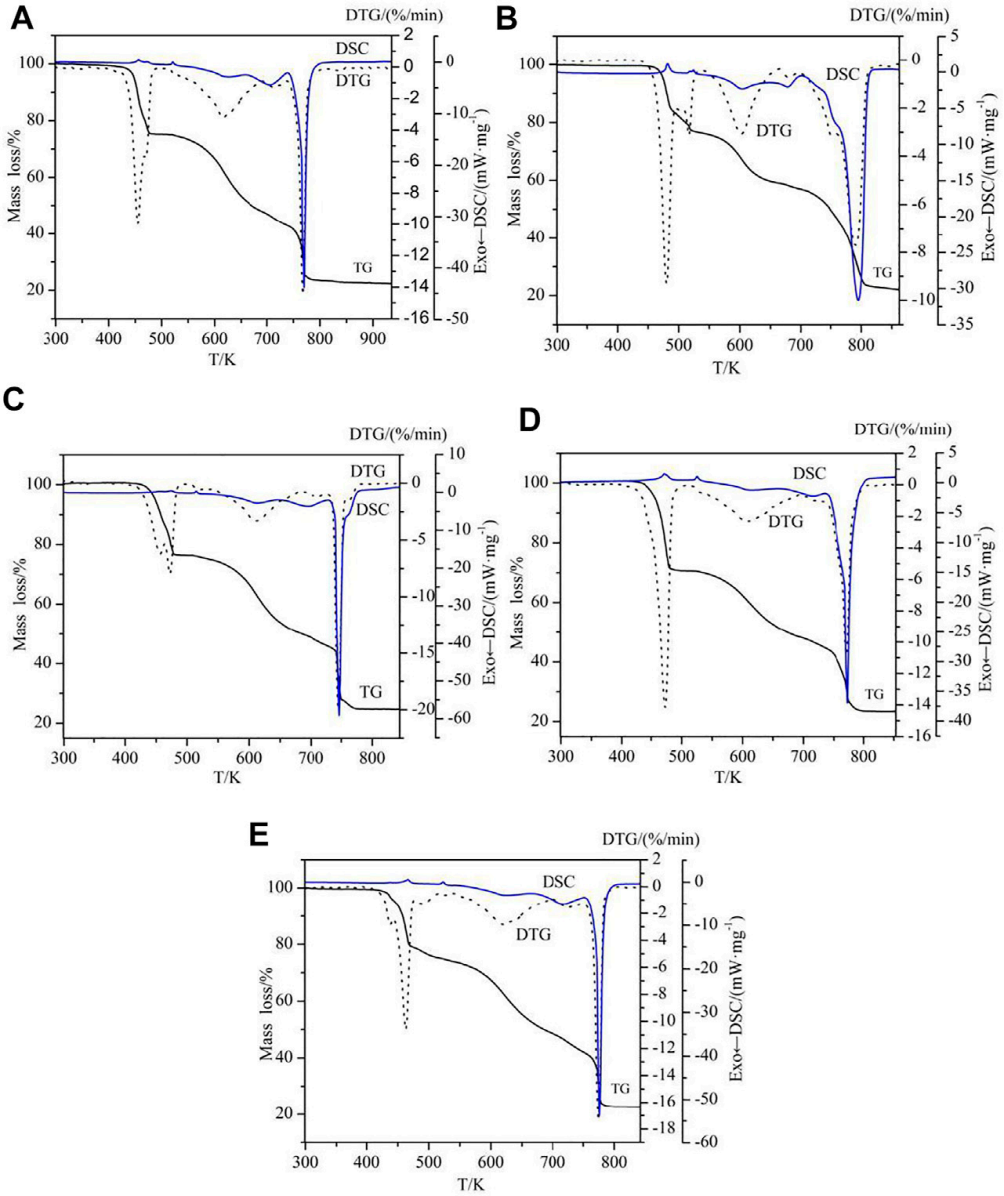
TG-DTG/DSC curves of the complexes **1**–**5** [**(A–E)** = complexes **1**–**5**].

To examine the thermal stability and thermal decomposition mechanism of the complexes **1**-**5**, the thermogravimetric analysis (TGA) were investigated. The TG-DTG-DSC curves are illustrated in Figure 9 and the thermal decomposition data are collected in Table 3. The complexes have three different structures, so complexes **2**, **3** and **4** are used as an example for further discussion.

**TABLE 3:** Mass loss pattern for the complexes **1**-**5**.

Complexes	Steps	Temperature range/K	DTG Tp/K	Mass loss rate (%)		Probable expelled groups	Intermediate and residue
				Found	Calcd		
1	I	345.15–500.15	455.05	25.42	26.92	4 (H_2_O) + 2 (5,5′-DM-2,2′-bipy)	Sm_2_ (2,4-DMBA)_6_
	II	500.15–691.15	615.75	31.59		x (2,4-DMBA)	Sm_2_ (2,4-DMBA)_6-x_
	III	691.15–935.15	767.75	21.03	51.77	6-x (2,4-DMBA)	Sm_2_O_3_
				78.04	78.69		
2	I	343.15–555.15	480.05	24.26	25.23	2 (H_2_O) + 2 (5,5′-DM-2,2′-bipy)	Eu_2_ (2,4-DMBA)_6_
	II	555.15–706.15	602.25	18.90		x (2,4-DMBA)	Eu_2_ (2,4-DMBA)_6-x_
	III	706.15–905.15	790.35	34.87	52.82	6-x (2,4-DMBA)	Eu_2_O_3_
				78.03	78.05		
3	I	403.15–520.15	472.45	24.82	24.96	0.5(C_2_H_5_OH) + 2 (5,5′-DM-2,2′-bipy)	Pr_2_ (2,4-DMBA)_6_
	II	520.15–687.15	610.35	26.04		x (2,4-DMBA)	Pr_2_ (2,4-DMBA)_6-x_
	III	687.15–844.15	745.05	25.19	53.33	6-x (2,4-DMBA)	Pr_6_O_11_
				77.52	78.29		
4	I	423.15–499.15	472.75	29.63	28.76	0.5 (2,4-DMBAH)+2.25 (5,5′-DM-2,2′-bipy)	Tb_2_ (2,4-DMBA)_6_
	II	499.15–700.15	607.95	22.72		x (2,4-DMBA)	Tb_2_ (2,4-DMBA)_6-x_
	III	700.15–853.15	771.55	24.61	49.28	6-x (2,4-DMBA)	Tb_4_O_7_
				76.96	78.01		
5	I	413.15–549.15	462.95	26.24	28.60	0.5 (2,4-DMBAH)+2.25 (5,5′-DM-2,2′-bipy)	Dy_2_ (2,4-DMBA)_6_
	II	549.15–701.15	619.55	24.93		x (2,4-DMBA)	Dy_2_ (2,4-DMBA)_6-x_
	III	701.15–842.15	774.65	25.93	49.57	6-x (2,4-DMBA)	Dy_2_O_3_
				77.10	78.17		

The thermal decomposition processes of complexes **1** and **2** are the same except that the number of water molecules lost at the beginning of the reaction is different, so the complex **2** as an example for discussion. In Figure 9B, the decomposition of complex **2** has undergone three stages, according to the three peaks of DTG curve. The first stage of decomposition occurs in 343.15–555.15 K, it is caused by two water and two 5,5′-DM-2,2′-bipy ligands, with a weight loss of 24.26%. The second decomposition took place in 555.15–706.15 K, and the weight loss rate was 18.90%. The theoretical value of loss of all 2,4-dimethylbenzoic acid ligands is 53.33%, which indicates that some 2,4-DMBA ligands are lost in this step. The last decomposition occurred at 706.15–905.15 K, corresponding to the decomposition of residual 2,4-DMBA ligands, and the weight loss rate was 34.87%. The total mass loss is 78.03%, and the final decomposition product is the metal oxide Eu_2_O_3_.

For complex **3**, its thermal decomposition process can be divided into three stages. The first stage is at 403.15–520.15 K, the weight loss is 24.82%, which corresponds to the loss of half an ethanol molecule and two 5,5′-DM-2,2′-bipy ligands. In the second stage at 520.15–687.15 K, the weight loss was 26.04%, which is attributed to the loss of part of 2,4-DMBA ligands. In the last stage at 687.15–844.15 K, the mass loss of the remaining 2,4-DMBA ligands was 25.19%. The total mass loss is 75.95%, which is in good agreement with the theoretical value. The final decomposition product is the metal oxide Pr_6_O_11_.

For complex **4**, its thermal decomposition process also can be divided into three stages. The first stage at 423.15–499.15 K, the weight loss rate is 29.63%, which is mainly ascribed to the loss of half free 2,4-DMBAH molecule, a quarter free 5,5′-DM-2,2′-bipy molecule and two 5,5′-DM-2,2′-bipy ligands. For the next two steps (the second and last), the total mass loss is 47.33%, which is attributed to the decomposition of six 2,4-DMBA ligands. The final product of the complete disintegration of complex **4** is Tb_4_O_7_. The total weight loss is 76.96%, close to the theoretical value.

### Evolved Gas Study During Thermal Decomposition

**FIGURE 10:**
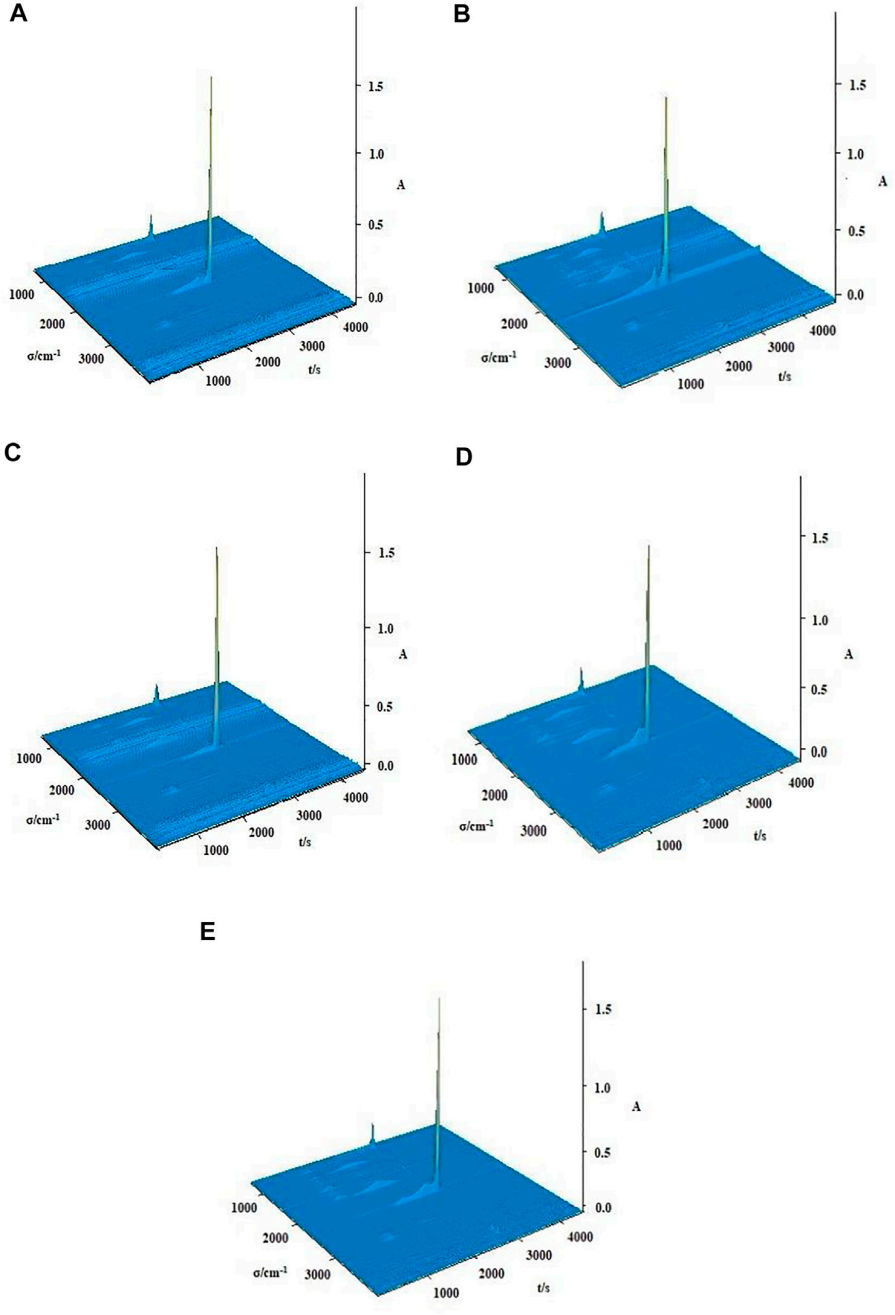
The 3D stacked plots of FTIR spectra of gaseous products during the thermal decomposition of the complexes **1**–**5** [**(A–E)** = complexes **1**–**5**].

**FIGURE 11:**
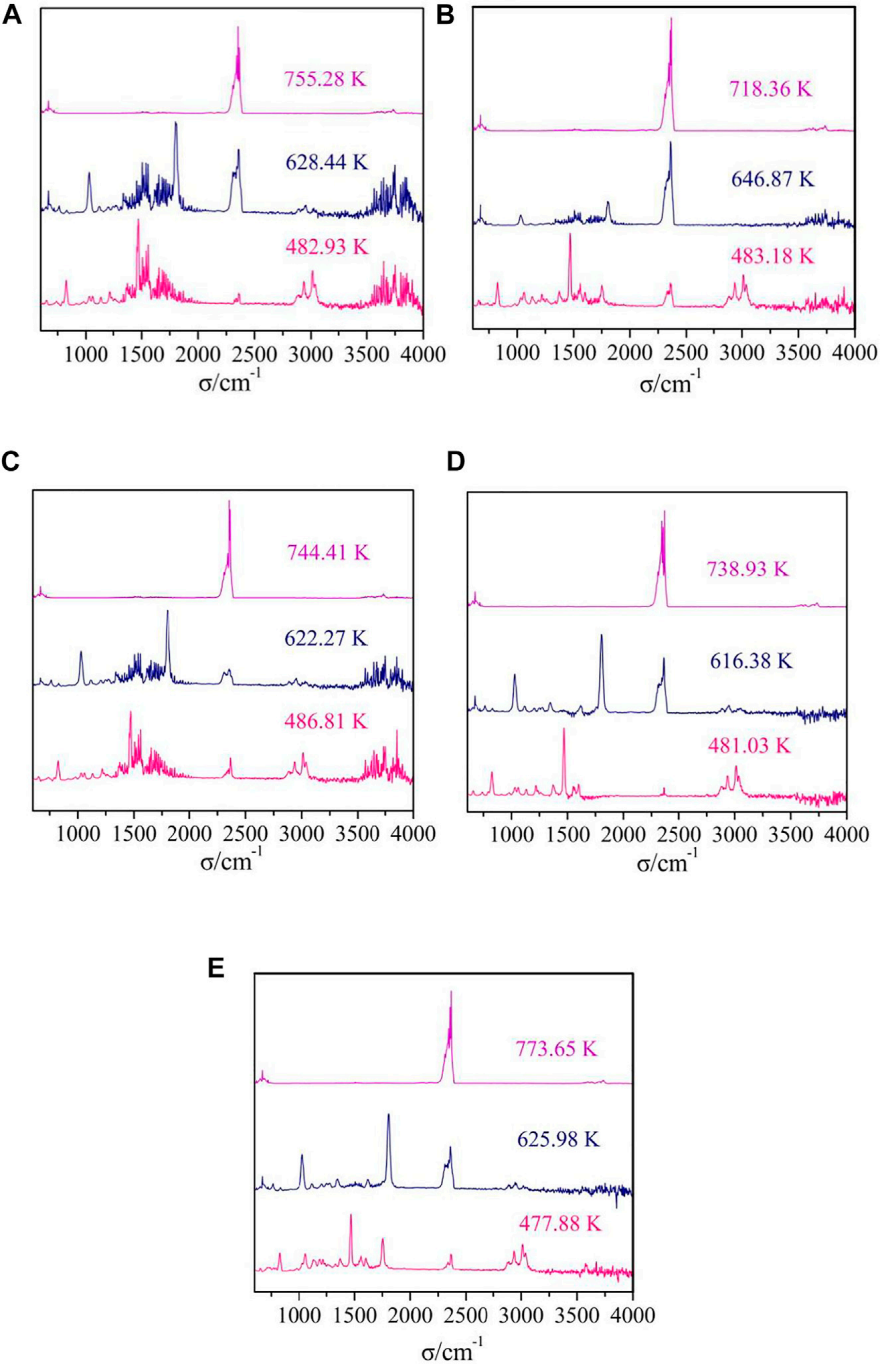
The FTIR spectra of the gaseous mixtures at different temperatures of complexes **1**–**5** [**(A–E)** = complexes **1**–**5**].

The TG-FTIR spectra of gaseous products of thermal decomposition processes for complexes **1**-**5** were obtained by TG/DSC-FTIR system in dynamic simulated air atmosphere. The 3D infrared spectra and 2D infrared spectra of complexes at different temperature are shown in Figures 10, 11. Similarly, complex **2**, complex **3** and complex **4** are used as an example for further discussion.

The difference between complex **1** and complex **2** is that the number of water molecules lost in the first stage of decomposition is different, but they have similar IR spectra of gaseous products. The gas released during the thermal decomposition of complex **2** can be divided into three characteristic absorption processes corresponding to the thermal decomposition process (Figure 11B). In the first infrared spectra (T = 483.18 K), the absorption bands of CO_2_ (2277–2405 cm^−1^, 658 cm^−1^) and the bands of H_2_O (3,644–3,910 cm^−1^) are observed. What’s more, some organic gaseous molecular fragments were found, such as ν_C=N_ (1,467 cm^−1^), ν_C-H_ (2828–3,121 cm^−1^), γ_=C-H_ (829 cm^−1^), ν_C=C_ (1,560 cm^−1^). These are mainly attributed to the decomposition of two water molecules and two 5,5′-DM-2,2′-bipy ligands. In the second infrared spectra (T = 646.87 K), we can observe the obvious strong absorption bands of CO_2_ (2233–2420 cm^−1^, 672 cm^−1^), and the low intensity absorption bands of H_2_O (3,566–3,881 cm^−1^). In addition, the absorption bands of some gaseous organic molecular fragments of 2,4-DMBA ligands, such as ν_C=O_ (1804 cm^−1^), ν_C=C_ (1,551 cm^−1^) are also detected. This also indicates that 2,4-DMBA ligands have begun to decompose in the stage. In the infrared spectra at 718.36 K, the bands of CO_2_ at 2241–2427 cm^−1^ and 672 cm^−1^ and the band of H_2_O at 3,559–3,787 cm^−1^ are observed. These results indicate that the ligands of 2,4-DMBA ligands has been completely decomposed.

For the complex **3**, there are three characteristic absorption processes in Figure 11C. In the first step, the stretching vibration absorption band of associated hydroxyl groups of C_2_H_5_OH molecules are observed at 3,855 cm^−1^. The characteristic bands of H_2_O (3,575–3,807 cm^−1^) and CO_2_ (2271–2422 cm^−1^, 653 cm^−1^) are observed. Additionally, some characteristic absorption bands such as ν_C=N_, ν_C-H_, γ_=C-H_, ν_C=C_, are also found in IR spectra. All these indicate that the ethanol molecules and 5,5′-DM-2,2′-bipy ligands are decomposed in this step. The next two steps are similar to that of complex **2**, corresponding to the decomposition of the 2,4-DMBA ligands.

For complex **4**, there are three characteristic absorption processes in Figure 11D. In the first step (T = 481.03 K), the absorption bands of H_2_O (3,551–3,887 cm^−1^) and CO_2_ (2320-2378 cm^−1^, 652 cm^−1^) are observed. Furthermore, the absorption bands of some small molecular fragments are found, such as ν_C=N_ (1,469 cm-1), ν_C-N_ (1,218 cm^−1^), ν_C-H_ (2856–2985 cm^−1^), ν_C=C_ (1,554, 1,603 cm^−1^), γ_=C-H_ (828, 1,032, 1,131 cm^−1^). This indicates that all neutral ligand 5,5′-DM-2,2′-bipy and free 2,4-DMBA ligands are lost in the first step of decomposition. The next two steps are similar to that of complex **2** and **3**, in which the 2,4-DMBA ligands are decomposed.

### Luminescent Property

**FIGURE 12:**
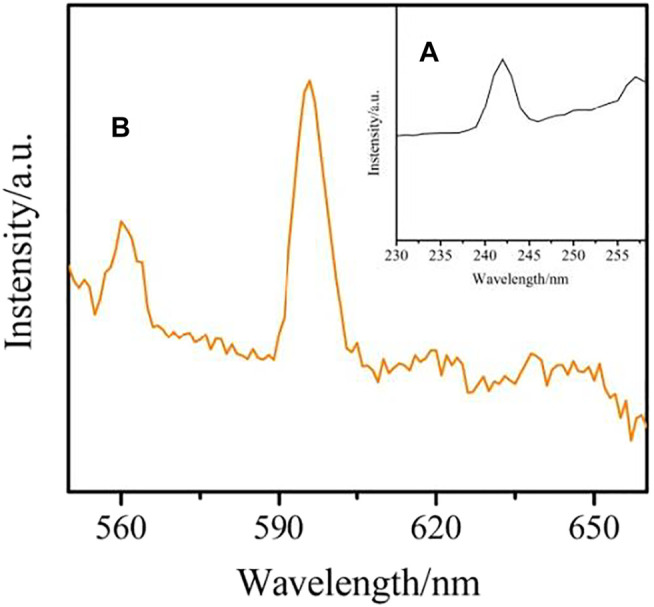
The Solid-state excitation **(A)** and emission **(B)** spectrum of complex **1**.

The solid state excitation and emission spectra complex **1-2** and **4** were obtained. The excitation spectrum of complex **1** are measured by monitoring the emission of Sm^3+^ ions at 596 nm is shown in Figure 12A. It exhibits a wide absorption band in the wavelength range of 235–245 nm, which is mainly attributed to the π→π* transition of the organic ligand. The emission spectrum of complex **1** also shows the characteristic peak of Sm^3+^ ion, which indicates that the organic ligand can sensitize the emission of Sm^3+^ ion. The emission spectrum of Sm^3+^ ion (λex = 242 nm) is illustrated in Figure 12B. The Sm^3+^ ion exhibits three characteristic peaks at 560 nm, 596 and 638 nm, corresponding to ^4^G_5/2_→^6^H_5/2_,^4^G_5/2_→^6^H_7/2_,^4^G_5/2_→^6^H_9/2_ transitions, respectively. The transition of ^4^G_5/2_→^6^H_7/2_ is stronger than the other, resulting in the characteristic orange-red luminescence of Sm^3+^ions (Carter et al., 2014; Chauhan and Langyan, 2020).

**FIGURE 13:**
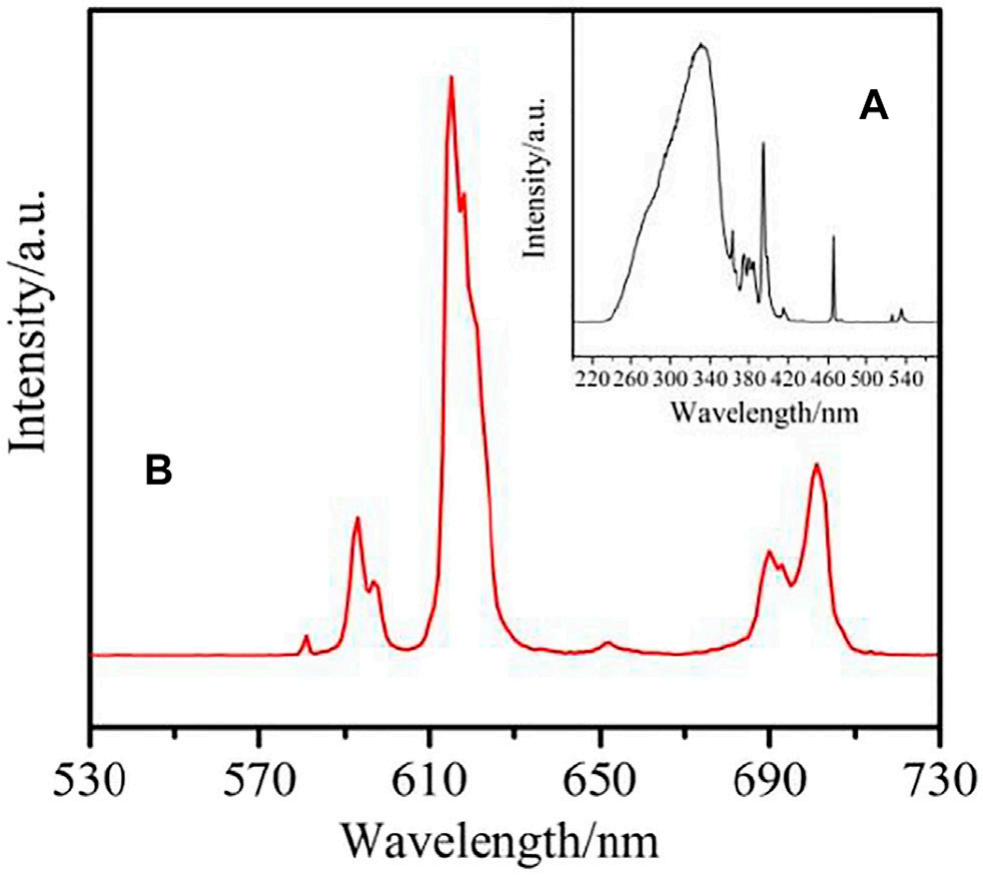
The Solid-state excitation **(A)** and emission **(B)** spectrum of complex **2**.

The excitation spectrum for complex **2** (Figure 13A), monitoring at λ_em_ = 615 nm, reveals a broad band at 220–380 nm referring to π→π* transition of the organic ligand in the complex. The emission spectrum of complex **3** is obtained upon excitation of 330 nm. The emission spectrum of complex **2** (Figure 13B) exhibits five characteristic transitions of Eu^3+^ ions: ^5^D_0_→^7^F_0_ (580 nm), ^5^D_0_→^7^F_1_ (592 nm), ^5^D_0_→^7^F_2_ (615 nm), ^5^D_0_→^7^F_3_ (653 nm), ^5^D_0_→^7^F_4_ (701 nm). The supersensitive transition of ^5^D_0_→^7^F_2_ at 615 nm is dominant in the whole spectrum, which is also the reason for the red light emission of the complex (Kataoka et al., 2014; Liu et al., 2020).

**FIGURE 14:**
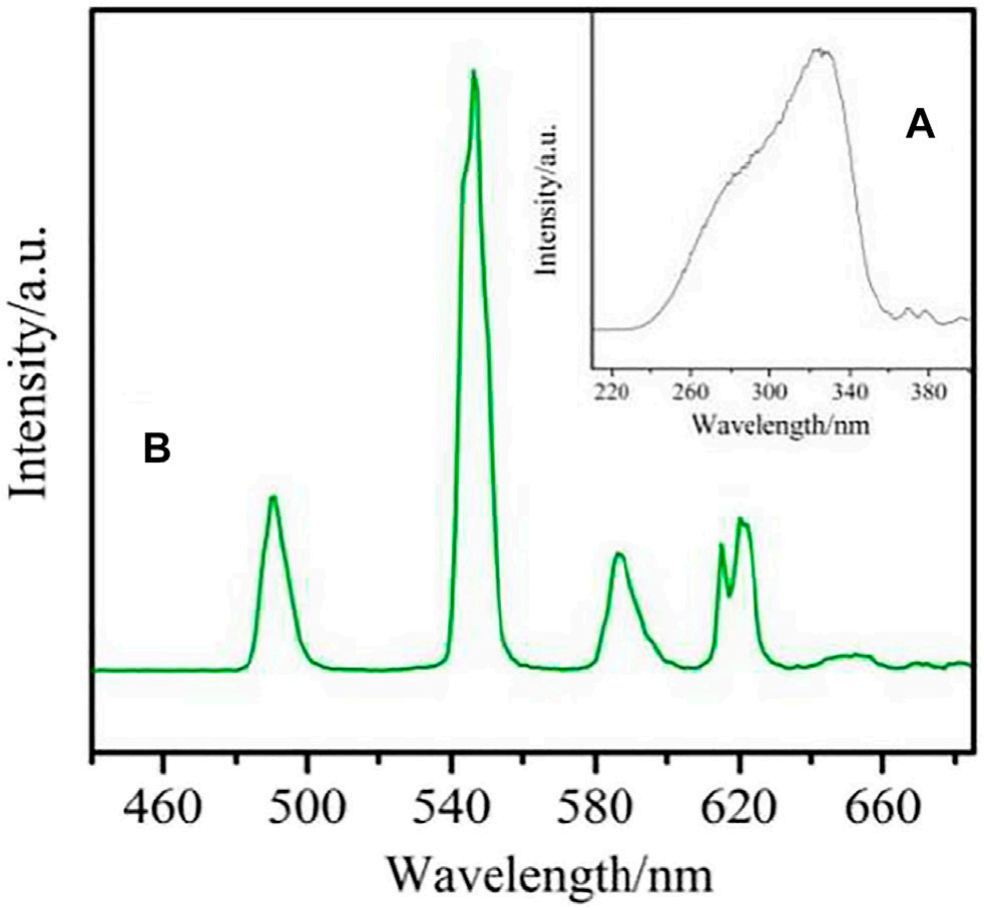
The Solid-state excitation **(A)** and emission **(B)** spectrum of complex **4**.

The excitation spectrum of complex **4** was obtained under emission at 546 nm (Figure 14A). Due to the electronic transition of organic ligands, it shows a wide band between 230–365 nm. It is also shown that the antenna effect is effective for the Tb(III) complex. The four emission peaks at 490 nm, 546 nm, 586 nm and 621 nm correspond to the ^5^D_4_→^7^F_J_ (j = 6→3) electronic transition of Tb^3+^ ion, and are obtained by excitation at 334 nm (Figure 14B). The transition of ^5^D_4_→^7^F_5_ at 546 nm controls the whole emission spectrum, which is the reason for the green emission of the complex (Batrice et al., 2017; Kot et al., 2019).

**FIGURE 15:**
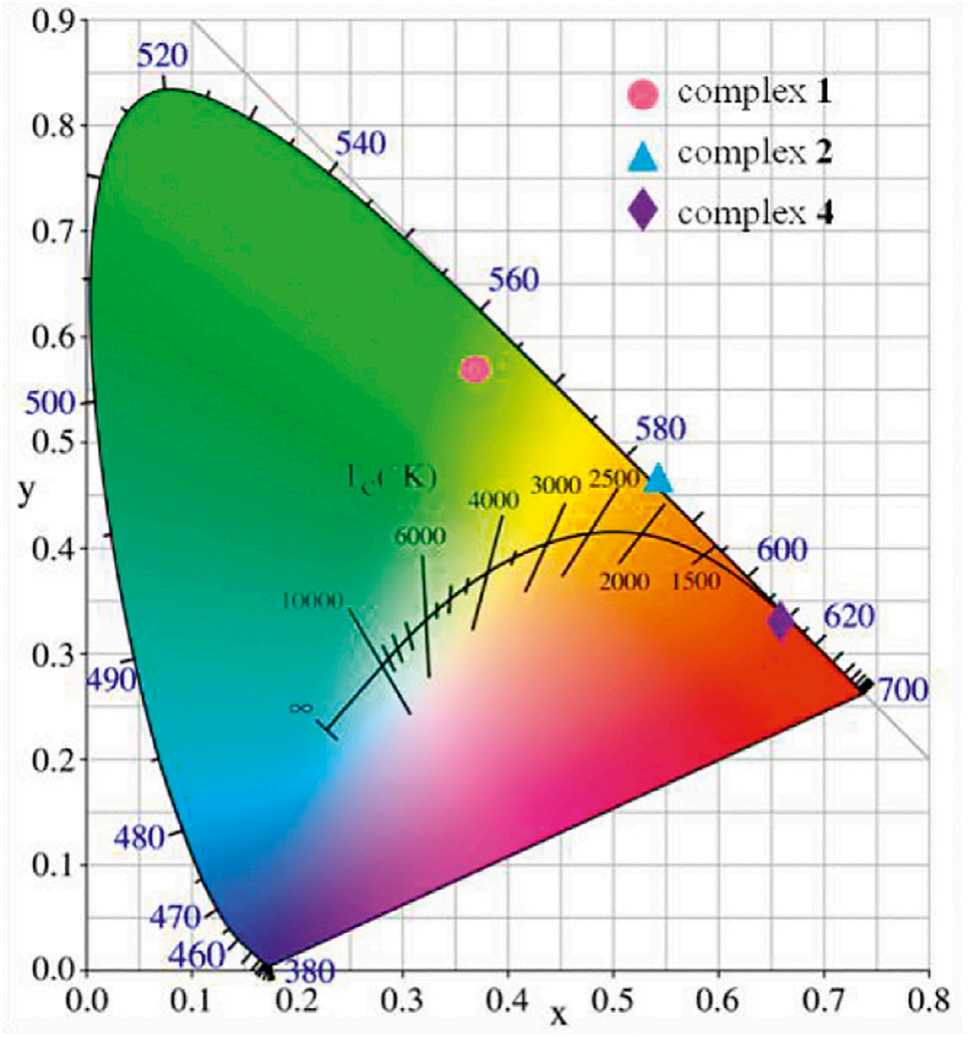
CIE chromaticity diagram presenting (x, y) color coordinate for complexes **1**-**2** and **4**.

The CIE chromaticity coordinates of complexes **1**-**2** and **4** are given in Figure 15. The emission data of complexes were calculated and marked by colored spots at (0.540, 0.458), (0.665, 0.332), (0.375, 0.564), respectively. Further analysis of CIE chromaticity coordinates indicates that complexes **1**-**2** and **4** is ideal candidate for orange-red, red and green component, respectively. These complexes may be very promising in the field of luminescent materials (Zhu et al., 2019).

### Luminescence Lifetime

**FIGURE 16:**
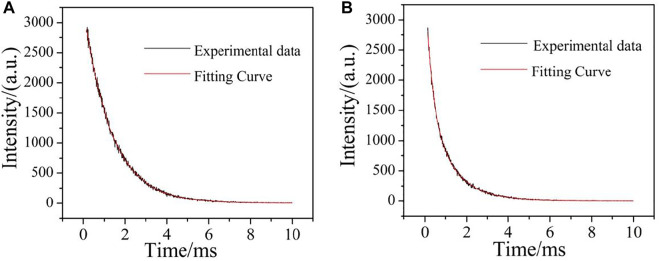
Decay and fitting curves for complexes **2** and **4** [**(A)**: complex **2**; **(B)** complex **4**].

The study of luminescence lifetime is also an important parameter to characterize the luminescent properties of fluorescent materials. The complexes containing Eu^3+^ and Tb^3+^ ions have strong photoluminescence properties and long luminescence lifetime (Li et al., 2020). Therefore, the luminescent lifetime of complexes **2** and **4** at room temperature was studied. The lifetime of the complex **2** was measured at the optimum excitation wavelength (330 nm) and emission wavelength (615 nm). As shown in Figure 16A, the luminescent decay curves were fitted by a double-exponential decay function. According to Eqs 1, 2 (Zhang et al., 2014), the luminescence lifetime can be calculated, i.e. 1.33 ms. The lifetime of complex **4** was determined at the optimum excitation wavelength (325 nm) and emission wavelength (546 nm). As shown in Figure 16B, the luminescence decay curve is fitted by a double exponential decay function. Similarly, according to the following formula, the luminescence lifetime is 1.01 ms.

$$I\left(t\right)=B_{1}\exp\left(\frac{-t}{\tau_{1}}\right)+B_{2}\exp\left(\frac{-t}{\tau_{2}}\right),$$ (1)

$$\tau =\frac{\left(B_{1}\tau_{1}^{2}+B_{2}\tau_{2}^{2}\right)}{\left(B_{1}\tau_{1}+B_{2}\tau_{2}\right)},$$ (2)

Where t is the time, I is the fluorescence intensity at time t, τ_1_ and τ_2_ are the decay times, and B1 and B2 are the fitting constants.

## Conclusion

In this article, these five lanthanide complexes have been successfully synthesized. The series of complexes have three different structural types. Complexes **1**-**2** are isomorphic and crystallized in the triclinic space group Pī. The coordination number is 9, and coordination environment is distorted monocapped square anti-prismatic geometry. Complex **3** crystallized in the monoclinic space group C2/c. Complex **3** and **1**-**2** have the same coordination number and environment. Complexes **4**-**5** are isomorphic and crystallized in the triclinic space group Pī, but their coordination number is 8 and their coordination environment is distorted tetragonal antiprism geometry. Complexes **1**–**3** form a one-dimensional chain structure, while complexes **4**-**5** form a two-dimensional network structure. The thermal behaviour of complexes are determined by TG-DSC/FTIR, the result indicate that the decomposition process of complexes are mainly divided into three stages and the final product is respective oxides. What’s more, the luminescence properties of complexes **1**-**2** and **4** were discussed, and calculated the luminescence lifetime (τ) of complexes **2** and **4**. These complexes may be potential fluorescent materials.

## Data Availability

The number of four complexes [CCDC 2051167 (1), CCDC 2051168 (2), CCDC 2051170 (3), CCDC 2051171 (4), CCDC 2051172 (5)] contains the supplementary crystallographic data for this paper. These data can be obtained free of charge from the Cambridge Crystallographic Date Centre via www.ccdc.cam.ac.uk/data_request/cif. Additional supporting information may be found online in the Supporting Information section at the end of this article.
