# Supplementary material for: Five Dinuclear Lanthanide Complexes Based on 2,4-dimethylbenzoic Acid and 5,5′-dimethy-2,2′-bipyridine: Crystal Structures, Thermal Behaviour and Luminescent Property
Source: Front Chem. 2021 Oct 18;9:726813. doi: 10.3389/fchem.2021.726813 (PMC8559305; doi:10.3389/fchem.2021.726813)
Supplement: Supplementary file 1 [file Table_1.DOCX]

**Table S1** The main absorption bands in the IR spectra for ligands and complexes **1**-**5** (cm^-1^).

| ligands/complexes | ***ν*_C=N_** | ***δ*_C-H_** | **ν_C=O_** | **ν_as(COO_^-^_)_** | **ν_s(COO_^-^_)_** | ***ν*_(Ln-O)_** |
| --- | --- | --- | --- | --- | --- | --- |
| 2,4-dimethylbenzoic acid |  |  | 1693 |  |  |  |
| 5,5′-dimethyl-2,2′-bipyridine | 1588 | 832 787 |  |  |  |  |
| **1** | 1523 | 834 788 |  | 1606 | 1412 | 419 |
| **2** | 1526 | 835 787 |  | 1605 | 1412 | 418 |
| **3** | 1523 | 835 787 |  | 1605 | 1411 | 418 |
| **4** | 1527 | 838 788 |  | 1615 | 1418 | 419 |
| **5** | 1528 | 839 788 |  | 1615 | 1422 | 419 |

**Table S2** Raman spectra data of ligands and complexes **1**-**5** (cm^-1^)

| ligands/complexes | ***ν*_C=N_** | ***δ*_C-H_** | **ν_C=O_** | **ν_as(COO_^-^_)_** | **ν_s(COO_^-^_)_** | ***ν*_(Ln-O)_** | ***ν*_(Ln-N)_** |
| --- | --- | --- | --- | --- | --- | --- | --- |
| 2,4-dimethylbenzoic acid |  |  | 1612 |  |  |  |  |
| 5,5′-dimethyl-2,2′-bipyridine | 1497 | 839 |  |  |  |  |  |
| **1** | 1379 | 847 |  | 1611 | 1501 | 331 | 257 |
| **2** | 1379 | 847 |  | 1602 | 1506 | 347 | 286 |
| **3** | 1382 | 842 |  | 1609 | 1506 | 346 | 295 |
| **4** | 1375 | 843 |  | 1601 | 1503 | 351 | 290 |
| **5** | 1381 | 847 |  | 1601 | 1503 | 325 | 291 |

**TableS3** The main bond lengths for complexes **1**-**2**.

| Complex 1 | Bond length/Å | Complex 2 | Bond length/Å |
| --- | --- | --- | --- |
| Sm(1)-O(7) | 2.393(12) | Eu(1)-O(5) | 2.335(9) |
| Sm(1)-O(5) | 2.410(13) | Eu(1)-O(7) | 2.346(11) |
| Sm(1)-O(3) | 2.421(12) | Eu(1)-O(3) | 2.349(11) |
| Sm(1)-O(2) | 2.449(13) | Eu(1)-O(9) | 2.395(12) |
| Sm(1)-O(10) | 2.468(15) | Eu(1)-O(2) | 2.421(11) |
| Sm(1)-O(9) | 2.472(14) | Eu(1)-O(10) | 2.425(10) |
| Sm(1)-N(2) | 2.630(16) | Eu(1)-N(1) | 2.522(13) |
| Sm(1)-N(1) | 2.660(17) | Eu(1)-N(2) | 2.554(14) |
| Sm(1)-O(1) | 2.714(14) | Eu(1)-O(1) | 2.688(11) |
| Sm(2)-O(1) | 2.363(12) | Eu(2)-O(6) | 2.333(10) |
| Sm(2)-O(6) | 2.402(12) | Eu(2)-O(1) | 2.341(11) |
| Sm(2)-O(8) | 2.405(12) | Eu(2)-O(11) | 2.371(11) |
| Sm(2)-O(11) | 2.430(14) | Eu(2)-O(8) | 2.379(9) |
| Sm(2)-O(12) | 2.509(14) | Eu(2)-O(12) | 2.426(11) |
| Sm(2)-O(4) | 2.538(13) | Eu(2)-O(4) | 2.475(11) |
| Sm(2)-O(3) | 2.605(14) | Eu(2)-N(3) | 2.521(12) |
| Sm(2)-N(3) | 2.636(15) | Eu(2)-N(4) | 2.563(12) |
| Sm(2)-N(4) | 2.656(15) | Eu(2)-O(3) | 2.638(10) |

**TableS4** The main bond lengths for complexes **3**-**5**.

| Complex 3 | Bond length/Å | Complex 4 | Bond length/Å | Complex 5 | Bond length/Å |
| --- | --- | --- | --- | --- | --- |
| Pr(1)-O(1) | 2.417(5) | Tb(1)-O(1) | 2.302(6) | Dy(1)-O(1) | 2.315(7) |
| Pr(1)-O(3)#1 | 2.429(4) | Tb(1)-O(3) | 2.337(6) | Dy(1)-O(3) | 2.334(6) |
| Pr(1)-O(2)#1 | 2.440(5) | Tb(1)-O(4)#1 | 2.340(5) | Dy(1)-O(4)#1 | 2.341(6) |
| Pr(1)-O(4) | 2.476(5) | Tb(1)-O(2)#1 | 2.373(5) | Dy(1)-O(2)#1 | 2.378(7) |
| Pr(1)-O(6) | 2.521(5) | Tb(1)-O(5) | 2.407(5) | Dy(1)-O(6) | 2.397(7) |
| Pr(1)-O(5) | 2.542(5) | Tb(1)-O(6) | 2.431(6) | Dy(1)-O(5) | 2.420(7) |
| Pr(1)-N(2) | 2.637(5) | Tb(1)-N(1) | 2.564(7) | Dy(1)-N(2) | 2.559(8) |
| Pr(1)-N(1) | 2.691(6) | Tb(1)-N(2) | 2.568(7) | Dy(1)-N(1) | 2.564(8) |
| Pr(1)-O(3) | 2.865(5) |  |  |  |  |
